# Spontaneous regression of tumours. Possible cross reactivity of autoantibodies against carbonic anhydrase I

**DOI:** 10.1111/jcmm.17970

**Published:** 2023-09-29

**Authors:** Ján Lakota

**Affiliations:** ^1^ Centre of Experimental Medicine, SAS Bratislava Slovakia

**Keywords:** aplastic anaemia‐like syndrome, carbonic anhydrase I, DNA polymerase theta, spontaneous tumour regression

## Abstract

Spontaneous tumour regression in patients after high dose therapy and autologous stem cell transplantation or patients with standard therapy is accompanied with the presence of high titers autoantibodies against carbonic anhydrase I (CA I). The concomitant presence of aplastic anaemia‐like syndrome in these patients points to parallel bone marrow suppression during this period. It seems that CA I, an ‘obscure’ enzyme, does not have any significant physiological role in humans. One possible explanation points to the fact that autoantibodies against CA I may target another antigen(s) which is(are) important in tumour growth as well as in normal haematopoiesis. One of the candidates for such a target is the DNA polymerase theta.

## 1 INTRODUCTION

Spontaneous tumour regression has been observed in almost all types of human cancer. The highest numbers of such cases have been reported in patients with lymphoma/leukaemia, malignant melanoma, neuroblastoma and renal cell carcinoma (‘hypernephroma’). The mechanism of spontaneous tumour regression is unknown. Some of the published data indicate that immune mediation, possible tumour growth inhibition by growth factors and/or cytokines, induction of tumour differentiation, hormonal action, tumour necrosis, psychological factors and epigenetic mechanisms may be involved in this process.^1^ An explanation of how and why malignant tumours undergo spontaneous remission without external treatment would lead to the possibility of improved methods of treating and preventing cancer.^2^, ^3^ We have described spontaneous regression of tumours after high dose therapy (HDT) and autologous stem cell transplantation (AuSCT). It has been associated with aplastic anaemia‐like syndrome.^4^ The sera of these patients were characterized by the presence of high titers of autoantibodies against carbonic anhydrase I.^5^ Here we present a possible explanation based on cross reactivity of the autoantibodies against carbonic anhydrase I.

## 2 SPONTANEOUS TUMOUR REGRESSION

**FIGURE 1:**
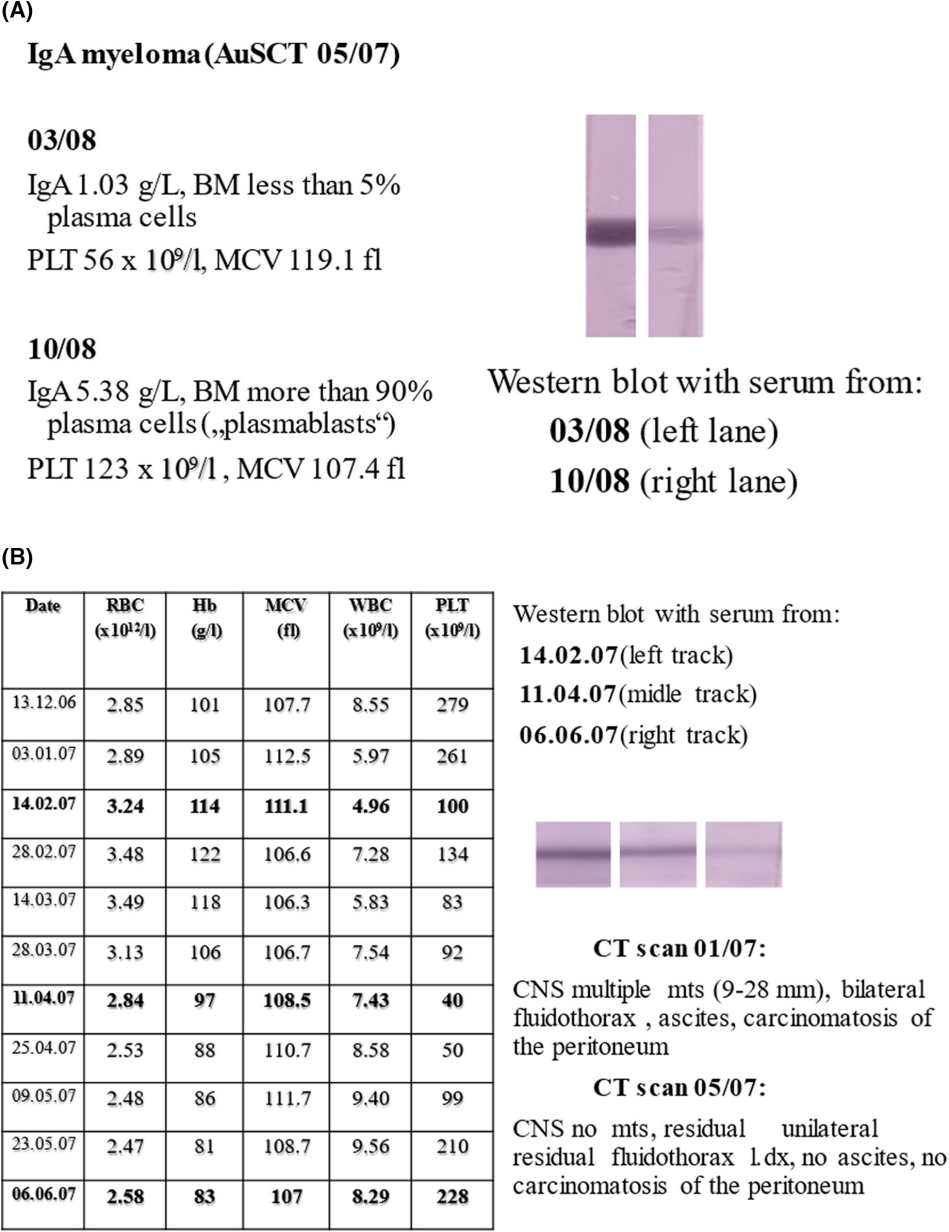
(A) Patient with IgA multiple myeloma treated with high dose therapy and autologous stem cell transplantation in May 2007. For further details please see the text. (B) Patient with metastatic breast carcinoma treated with conventional therapy. For further details please see the text.

Spontaneous tumour regression after its relapse after HDT and autologous stem cell and AuSCT has been firstly described in 2003.^4^ During the period of spontaneous regression, the patients' blood counts strongly resembled the blood pattern of patients with bona fide aplastic anaemia (AA). Moreover, these patients' morphology of the bone marrow trephine biopsies was identical to the AA picture. Therefore, this syndrome was classified as aplastic anaemia (AA)‐like syndrome. Later, Nissen and Stern proposed that the autoimmune (anti‐tumour) activity against present malignancy could operate against haematopoietic stem cells in the bone marrow.^6^ This pathophysiological mechanism could explain bicytopenia or pancytopenia. This phenomenon is frequently present in patients with haematological as well as non‐haematological malignancies. The explanation would be that pancytopenia which is present in AA or AA‐like syndrome reflects an ongoing immune reaction against the underlying malignancy. By analysis of the sera of patients with spontaneous tumour regression and (AA)‐like syndrome, it has been shown that they contain antibodies against carbonic anhydrase I (anti‐CA I antibodies).^5^ These antibodies were polyclonal. Mapping of CA I enzyme (CA I), four linear immunodominant epitopes of CA I enzyme (DGLAV, NVGHS, SLKPI and SSEQL) were detected.^7^ The presence of anti‐CA I autoantibodies strongly resembles the patient's blood pattern and the presence of her/his tumour. In Figure 1A, the patient with multiple IgA myeloma was treated with HD and AuSCT in May 2007. The patient remained bicytopenic (anaemia and thrombopenia) with high erythrocyte mean corpuscular volume (119.1 fL) for more than 10 months after AuSCT. The patient's myeloma was in complete remission. The presence of high titers anti‐CA I autoantibodies is shown on the Western blot left track. Seventeen months after AuSCT a fulminant relapse occurred. At that time the anti‐CA I autoantibodies practically disappeared (Western blot right track) and the blood counts were almost normal. The phenomenon of spontaneous tumour regression has been mainly observed in patients who relapsed after HDT and AuSCT. Nevertheless, albeit rather rarely, it has been observed in patients who were treated only with conventional chemotherapy. An example of such a patient with metastatic breast carcinoma is shown in Figure 1B. In January 2007 the patient presented as an emergency with metastases in the CNS. Palliative radiotherapy on CNS was applied. Suddenly the patient became bicytopenic (anaemia, thrombopenia) and was not eligible for any chemotherapy. As one can see the bicytopenia was accompanied with the presence of anti‐CA I autoantibodies in the patient's serum. In May 2007 the CT scan showed the picture of a complete remission of the malignant disease. The patient's blood counts normalized and concomitantly the anti‐CA I autoantibodies diminished and disappeared in June 2007. Sadly, the patient relapsed later in the same year 2007 and died on the progression of the disease.

## 3 BONA FIDE APLASTIC ANAEMIA

The anti‐CA I autoantibodies were found in patients with bona fide aplastic anaemia (AA).^8^ The antibodies were detected in 38% of analysed patients. The presence of antibodies was associated with worse prognosis. The complete response to anti‐thymocyte globulin treatment in antibody positive patients was 14% in contrast to 64% in the antibody negative patients. The antibody positive patients had an inferior 10‐year survival—36% in contrast to 64% in anti‐CA I negative patients. Moreover, these anti‐CA I autoantibodies in patients with bona fide AA recognize the same immunodominant linear CA I epitopes (NVGHS, DGLAV, SSEQL and SLKPI) as those anti‐CA autoantibodies in the sera of a patients, who developed AA‐like syndrome and spontaneously regressed after HDT and AuSCT.^9^

## 4 CROSS REACTIVITY—A POSSIBLE EXPLANATION?

The presence of anti‐CA I autoantibodies in the patient sera during the spontaneous regression of their tumours is striking. It is associated with the suppression of bone marrow, a phenomenon which got a classification ‘aplastic anemia‐like syndrome’ (‘AA‐like syndrome’). The anti‐CA I autoantibodies are present in up to 40% of patients in bona fide AA too.^8^ The immunodominant epitopes overlap in both patients' groups.^9^ The cross reactivity of patients' anti‐CA I auto antibodies against other members of the human carbonic anhydrases (including CA IX and CA XII) has been never observed on Western blots (unpublished data). CA I is an ‘obscure’ enzyme. In a ‘historical’ paper Kendall and Tashian^10^ describe a family from the Greek island Icaria with virtually absent CA I (0.6–0.7 ng CA I/mg haemoglobin in contrast to normal values 11.57 ± 2.26 μg CA I/mg haemoglobin) with any clinical consequences for members of the family. In other words, the CA I albeit the second most abundant protein in human erythrocytes seems to have no (or up to date any known) physiological role in humans.^11^ Nevertheless, it should be noted that CA I could be partially involved in the process of pH homeostasis, respiration and in erythroid differentiation, albeit its function is not in humans ‘vital’. The CAI is also linked to some pathological processes such as anaemia, chronic acidosis, diabetic macular oedema, proliferative diabetic retinopathy and vasogenic oedema.^12^, ^13^ Moreover, CA I seems to be a potential biomarker for some cancer types such as colorectal cancer,^14^ non‐small cell lung cancer^15^ and prostate cancer.^16^ On the other hand, anti‐CA I antibodies were described in some autoimmune diseases such as autoimmune/idiopathic chronic pancreatitis, Sjögren's syndrome,^17^ connective tissue diseases,^18^ systemic lupus erythematosus (SLE) and other rheumatic diseases. Furthermore, for some autoimmune diseases, anti‐CA I antibodies represent a predictable diagnostic marker.^19^ It should be noted that anaemia is the most frequent haematological abnormality in SLE. It is present in 63.0% patients.^20^ Interestingly, the suppression of haematopoiesis in some patients with SLE is mediated by IgG autoantibodies.^21^

**FIGURE 2:**
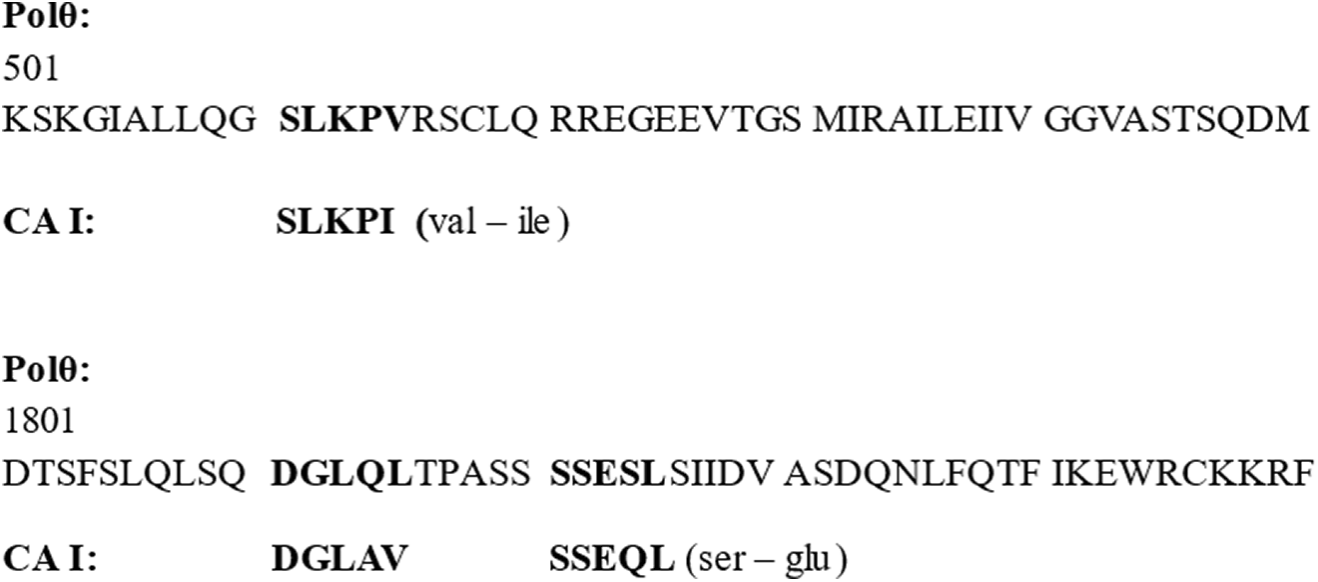
Possible ‘alignment of epitopes’ (Polθ, CA I). The initial amino acid of the string (Polθ) is K (541) and D (1801), respectively.

Therefore, it is tempting to search for other models which could explain the observed data obtained in laboratory experiments. One of the candidates with strong possible cross reactivity could be the enzyme DNA polymerase theta (Polθ). The DNA polymerase Polθ has been described rather in detail.^22^, ^23^, ^24^ From four established linear immune epitopes in CA I (DGLAV, NVGHS, SLKPI and SSEQL), the alignment with amino acid sequence of Polθ (https://www.uniprot.org/uniprotkb/O75417/entry#sequences) can be found in three of them: SLKPI, DGLAV and SSEQL. The almost full alignment with SLKPI sequence is at the Polθ position 511‐515 (SLKPV), with the DGLAV sequence at the Polθ position 1811‐1815 (DGLQL) and finally with the SSEQL sequence at the Polθ position 1821‐1825 (SSESL) (Figure 2). In the most stringent alignment, the Polθ SLKPV sequence contains amino acid valine instead of isoleucine of the CA I SLKPI sequence. The same stringency is present in the Polθ SSESL sequence which differs in the amino acid serine in contrast to glutamine of the CA I SSEQL sequence. Both valine and isoleucine are aliphatic and hydrophobic. On the other hand, serine, and glutamine which both are relatively polar. In the Polθ sequence DGLQL the full alignment with the CA I sequence DGLAV is in three amino acids DGL. However, QL (glutamine and leucine) share a lot of ‘common’ chemical properties with AV (alanine and valine). It should be noted that the amino acid sequence 511‐515 is in the Polθ helicase‐like domain, whereas the amino acid sequences 1811‐1815 and 1821‐1825 are found in the Polθ polymerase domain.^25^ The reverse transcriptase activity of Polθ has been shown in elegant experiments in Richard Pomerantz's lab.^26^ Therefore, Polθ seems to be an ideal candidate for a cancer drug target.^27^ Moreover, under physiological conditions, lymphoid tissue and bone marrow are the highest expressing human tissues of Polθ (https://www.proteinatlas.org/ENSG00000051341‐POLQ/tissue). From this point of view the pathophysiological explanation for bone marrow suppression (AA‐like syndrome and bona fide AA) and spontaneous tumour regression may have a common point: The suppression of the DNA polymerase Polθ which is vital for both bone marrow (haematopoiesis) and tumour cell growth. Clinically this resembles the picture of spontaneous tumour regression which is associated with AA‐like syndrome.

## AUTHOR CONTRIBUTIONS

**Ján Lakota:** Conceptualization (equal); data curation (equal); formal analysis (equal); funding acquisition (equal); investigation (equal); methodology (equal); writing – original draft (equal); writing – review and editing (equal).

## CONFLICT OF INTEREST STATEMENT

The author declares no conflict of interest.

## Data Availability

The data that support the findings of the study are available from the corresponding author upon reasonable request.
